# Einfluss verschiedener automatisierter externer Defibrillatoren auf die Hands-off-Intervalle von Ersthelfern

**DOI:** 10.1007/s10049-022-01059-z

**Published:** 2022-07-05

**Authors:** Volker Schäfer, Patrick Witwer, Lisa Schwingshackl, Hannah Salchner, Lukas Gasteiger, Wilfried Schabauer, Wolfgang Lederer

**Affiliations:** Medizinische Universität Innsbruck, Univ.-Klinik für Anästhesie und Intensivmedizin, Anichstr. 35, 6020 Innsbruck, Österreich

**Keywords:** Kardiopulmonale Reanimation, Defibrillation, Laienreanimation, Prähospitaler Herz-Kreislauf-Stillstand, Wiederbelebung, Cardiopulmonary resuscitation, Defibrillation, Bystanders CPR, Out-of-hospital cardiac arrest, Resuscitation

## Abstract

**Hintergrund:**

Die Überlebenschancen nach prähospitalen Herz-Kreislauf-Stillständen mit hyperdynamen elektrischen Herzrhythmen können durch frühzeitige Defibrillation mit einem automatisierten externen Defibrillator signifikant verbessert werden. Entsprechend den internationalen Leitlinien dürfen die daraus resultierenden Hands-off-Intervalle nicht mehr als 10 s betragen.

**Ziel der Arbeit:**

Wir untersuchten die Verzögerung des Beginns der Herzdruckmassage und die Dauer der Hands-off-Intervalle während der Reanimation, die mit der Anwendung von automatisierten externen Defibrillatoren in Zusammenhang stehen.

**Material und Methoden:**

In einer prospektiven, randomisierten, einfach verblindeten Beobachtungsstudie wurden Reanimationsmaßnahmen von Medizinstudent*Innen im ersten Semester in verschiedenen Notfallszenarien am Phantom getestet. Verglichen wurden die Verzögerung des Beginns der Herzdruckmassage und die Dauer der Hands-off-Intervalle durch Sprachanweisungen von vier konventionellen Geräten bei defibrillierbaren und nichtdefibrillierbaren Rhythmen. Zufriedenheit mit dem Gerät, Schwierigkeiten bei der Anwendung und Verbesserungsvorschläge wurden über Fragebogen erfasst.

**Ergebnisse:**

In insgesamt 70 Anwendungen wurde der Beginn der Herzdruckmassage durch die Sprachanweisungen um durchschnittlich 115 s verzögert. Bei defibrillierbaren Rhythmen wurde der erste Schock im Mittel erst nach 125 s ausgelöst. Unterbrechungen nach Schockabgabe von unter 10 s wurden mit keinem der getesteten Geräte erreicht. Die Hands-off-Intervalle während der Defibrillationen unterschieden sich signifikant zwischen den Geräten (*p* < 0,001). Verbesserungsvorschläge wurden für die Bereiche Gerätemarkierungen, Sprachanweisungen und Elektroden gemacht.

**Diskussion:**

Perischockpausen von unter 10 s konnten mit keinem der getesteten Geräte erreicht werden. Kürzere und konkretere Sprachanweisungen sowie übersichtlichere Beschriftung und Anordnung der Elektroden sind nötig, um die Anwendung zu vereinfachen, den Beginn der Herzdruckmassage weniger zu verzögern und die Hands-off-Intervalle zu verkürzen.

## Abstract (en)

**Background:**

Survival chances after out-of-hospital cardiac arrests caused by hyperdynamic electric cardiac rhythms can be significantly improved by early defibrillation with automated external defibrillators (AEDs). As postulated in international guidelines, the resulting hands-off intervals should not exceed 10 s.

**Objectives:**

We investigated delay in onset of chest compressions and the length of hands-off intervals during defibrillation associated with the application of AEDs.

**Materials and methods:**

In a prospective, randomized, single-blinded observational study, the resuscitation efforts by first year medical students were analyzed in different emergency scenarios on manikins. Delay in onset of chest compressions and the length of hands-off intervals between voice prompts from four conventional devices were compared during shockable and nonshockable rhythms. Satisfaction with the device, difficulties with the application, and suggested improvements were assessed by questionnaire.

**Results:**

In a total of 70 applications, the start with thoracic compressions was delayed by a mean of 115 s. On average, the first shock was administered after 125 s in shockable heart rhythms. Perishock pauses of less than 10 s were achieved with none of the tested devices. Hands-off intervals during defibrillation differed significantly between the devices (*p* < 0.001). Improvements were suggested regarding marking, voice prompts, and electrodes.

**Conclusions:**

Perishock pause of less than 10 s was not achieved with any of the tested devices. Shortened and more precise voice prompts as well as more clearly arranged labeling and layout of pads are needed to simplify application, reduce delayed onset of chest compressions and shorten hands-off intervals.

## Kurze Hinführung zum Thema

Die Verwendung von automatisierten externen Defibrillatoren (AED) innerhalb von 5 min nach prähospitalem Herz-Kreislauf-Stillstand durch Ersthelfer kann zu Überlebensraten von bis zu 70 % führen [2, 19]. Durch optimierte AED-Anwendung können sogar noch bessere Ergebnisse erzielt werden [9]. Das alleinige Verkürzen der Wartepausen während der Analyse, der Ladephase und der Schockanweisung ist dabei nicht ausreichend [1]. Es braucht eine ausgewogene Verbindung von klaren und präzisen Angaben mit vereinfachten Abläufen.

## Einführung

Öffentlich zugängliche automatisierte externe Defibrillatoren (AED) können von Ersthelfern für die kardiopulmonale Reanimation (CPR) bei prähospitalem Herz-Kreislauf-Stillstand verwendet werden, da sie Beginn und Durchführung von lebensrettenden Maßnahmen über AED-Sprachanweisungen deutlich erleichtern [7, 11]. Die aktuellen Standorte der AED können online oder via App abgefragt werden und sind auch über laufend aktualisierte Register von Disponenten der Rettungsleitstellen abrufbar [10].

AED-Sprachanweisungen unterscheiden sich je nach Produkt und Hersteller. Ob längere und detailliertere Anweisungen zu weniger Fehlern und besseren Reanimationsleistungen von Laien führen und damit Hands-off-Intervalle (HOI) ausgleichen können, ist nicht bekannt [17]. Da lange HOI zu schlechteren Überlebenschancen führen, müssen AED-Sprachanweisungen kurz und eindeutig sein und dabei trotzdem ausreichend Information bieten. Ersthelfer mit wenig Erfahrung wünschen sich eher ausführlichere Sprachanweisungen [20]. Das erfordert die Anpassungen der Texte an die Bedürfnisse der Benutzer bei gleichzeitiger Präzisierung der Textlänge [21]. Um eine Kontinuität der Reanimationsmaßnahmen zu ermöglichen, sollte ein rasches Aufeinanderfolgen von Sprachanweisungen vermieden werden [6].

In dieser Studie wurden die Umsetzbarkeit von AED-Sprachanweisungen durch Ersthelfer und die möglichen Auswirkungen auf die Maßnahmen während einer CPR am Phantom untersucht. Primäres Ziel war es festzustellen, welche Verzögerungen der Wiederbelebungsmaßnahmen bei der AED-Anwendung durch Ersthelfer bestehen. Speziell wurde analysiert, ob Unterbrechungen während der Schockabgabe durch AED-Sprachanweisungen innerhalb der zeitlichen Vorgaben der internationalen Leitlinien der Reanimation von 2015 und 2020 liegen [18, 19]. Sekundäres Ziel war es, Dauer und Klarheit der Sprachanweisungen zwischen verschiedenen AED zu vergleichen. Tertiäres Ziel war es, die Anwenderzufriedenheit zu erfragen und eventuelle Verbesserungsvorschläge einzuholen.

## Material und Methoden

Eine prospektive, randomisierte und einfach verblindete AED-Anwenderstudie wurde in den Schulungsräumen der Medizinischen Universität Innsbruck durchgeführt. Als Ersthelfer wurden Medizinstudent*Innen im ersten Semester eingeschlossen. Die Teilnahme war freiwillig und erfolgte noch vor dem verpflichtenden Basic-Life-Support(BLS)-Kurs im ersten Studienabschnitt. Die Einverständniserklärung erfolgte nach schriftlicher Information über den wissenschaftlichen Hintergrund der Studie unter dem Hinweis, dass die Ergebnisse anonymisiert in einem wissenschaftlichen Journal veröffentlicht werden. Nach Überprüfung auf Unbedenklichkeit durch die Ethikkommission der Medizinischen Universität Innsbruck (EK Nr.: 1165/2017) wurde diese Studie nach den Richtlinien der Helsinki-Erklärung durchgeführt [12].

Einschlusskriterien waren: freiwillige Teilnahme, Erstsemestrige im Human- oder Zahnmedizinstudium vor Absolvieren des BLS-Kurses. Vorkenntnisse, beispielsweise Erfahrung in der Krankenpflege oder im Rettungsdienst, wurden erfasst und als Untergruppe analysiert.

Ausschlusskriterien waren: Ablehnung oder vorzeitiger Abbruch der Teilnahme.

Da die Untersuchungen im Wintersemester 2017/2018 noch vor der COVID-19-Pandemie durchgeführt wurden, war auch die Beatmung unter Verwendung eines Beatmungstuchs zum Selbstschutz Teil der Ersthelfermaßnahmen. Es gab keine Interessenkonflikte zwischen den Durchführenden der Studie, den Anwendern und den Herstellern der AED.

### Studiendesign

In drei realitätsnahen Notfallszenarien wurde die Anwendung von vier handelsüblichen, frei erhältlichen AED an Reanimationspuppen (Resusci Anne Simulator, LAERDAL Medical AS, Stavanger, Norwegen) untersucht. Jedem Teilnehmer wurde vor Beginn der Untersuchung am Phantom eines von vier AED-Geräten und eines von drei Notfallszenarios randomisiert zugeteilt, indem aus einem Behälter ein Briefumschlag mit einem Notfallszenario und aus einem zweiten Behälter ein Briefumschlag mit dem AED-Typ gezogen wurde. Die Reanimationsszenarien waren stets als „Einhelferszenarien“ definiert.

Bei den drei Reanimationsszenarien unterschied sich die Sequenz des defibrillierbaren (Kammerflimmern) und des nichtdefibrillierbaren (Asystolie) Herzrhythmus. In Szenario A war der erste Herzrhythmus ein Kammerflimmern, der zweite eine Asystolie. In Szenario B zeigte die erste Analyse Asystolie, die zweite Kammerflimmern. In Szenario C wurde bei der ersten und zweiten Analyse Kammerflimmern simuliert.

Phantom und AED waren immer gleich angeordnet, ebenso der Abstand der Teilnehmer zum Phantom am Beginn der Untersuchung. Nachdem ein standardisierter Text zur Reanimationssituation vorgelesen worden war, begann die Zeitmessung. Die Zeitintervalle ohne Reanimation wurden als Verzögerung des Beginns der Herzdruckmassage und als HOI während der CPR erfasst. Im Speziellen wurden die HOI, die sich aus der Dauer der Sprachanweisung (systemimmanente Verzögerung) und der Reaktionszeit bis zum Beginn der Ausführung (anwenderbedingte Verzögerung) ergibt, ausgewertet. Primärer Endpunkt der Studie war es festzustellen, ob die Hands-off-Intervalle während der Defibrillation unter 10 s gehalten werden können, so wie es in den internationalen Leitlinien gefordert wird. Sekundäre Endpunkte der Studie waren die Dauern der Hands-off-Intervalle nach jeder Sprachanweisung sowohl beim inital defibrillierbaren als auch beim nichtdefibrillierbaren Herzrhythmus. Tertiärer Endpunkt war, die Benutzerzufriedenheit zu erheben und mögliche Verbesserungsvorschläge zu erfassen. Zusätzlich wurde festgehalten: Durchführung eines Notfallchecks, das Absetzen eines Notrufs, die Verwendung eines Beatmungstuchs zum Selbstschutz, ob die Reanimationspuppe während der Herzrhythmusanalyse berührt wurde und ob der Elektrodenkontakt fehlerhaft war.

Nach der Untersuchung wurden Alter, Geschlecht und Erfahrungsgrad der Teilnehmer erfasst. In einem kurzen Fragebogen wurden die Verständlichkeit der Sprachanweisungen und die Leichtigkeit der AED-Anwendbarkeit mit einer 5‑stelligen Likert-Skala (1 = sehr gut, 2 = gut, 3 = zufriedenstellend, 4 = ausreichend, 5 = nicht ausreichend) erfragt [15].

Etwaige Verbesserungsvorschläge wurden erhoben.

### AED-Geräte und Messintervalle

Die folgenden AED wurden verwendet:

Heartstart FR3 und Heartstart HS1 (FR3 und HS1; PHILIPS N.V. Healthcare, Amsterdam, Niederlande), Fred easyport® (FE; SCHILLER AG, Baar, Schweiz) und Lifepak® 1000 (LP; PHYSIO CONTROL, Redmond, USA).

Die verschiedenen Zeitpunkte der Messungen zur Evaluierung der unterschiedlichen AED-Typen wurden definiert als:

- T1: Start
- T2: Öffnen des AED (Reiß‑/Klettverschluss)
- T3: Beginn Sprachanweisung (Elektroden aufkleben)
- T4: Beginn Tätigkeit (Elektrodenpackung berührt)
- T5: Ende Tätigkeit (Elektroden aufgeklebt und einsatzbereit)
- T6: Beginn Sprachanweisung (1. Analyse)
- T7: Beginn Sprachanweisung (Schock oder CPR empfohlen)
- T8: Beginn Tätigkeit (Schock oder CPR)
- T9: Beginn Sprachanweisung (2. Analyse)
- T10: Beginn Sprachanweisung (Schock oder CPR empfohlen)
- T11: Beginn Tätigkeit (Schock oder CPR)

Daraus ergaben sich folgende Zeitintervalle:

- T1–T5: Zeit vom Start bis zum Aufkleben der Elektroden, also bis zur AED-Einsetzbarkeit. T1–T6: Zeit vom Start bis zum Start der ersten Sprachanweisung
- T1–T8: Zeit vom Start bis zur ersten Schockabgabe bzw. Beginn der Herzdruckmassage
- T6–T7: Dauer der ersten Herzrhythmusanalyse
- T6–T8: Zeit vom Beginn der ersten Herzrhythmusanalyse bis zur ersten Schockabgabe bzw. Beginn der Herzdruckmassage
- T7–T8: Zeitdauer zwischen der Sprachanweisung und der tatsächlichen Durchführung der Maßnahme (Schockabgabe bzw. Durchführung der Herzdruckmassage)
- T9–T10: Dauer der zweiten Herzrhythmusanalyse
- T9–T11: Zeitintervall vom Start der zweiten Herzrhythmusanalyse bis zur Schockabgabe oder zum Start der Herzdruckmassage
- T10–T11: Zeit von der zweiten Sprachanweisung zur Schockabgabe oder zur Durchführung der Herzdruckmassage bis zur tatsächlichen Ausführung der Maßnahme

### Statistik

Nullhypothese: Hands-off-Intervalle unterscheiden sich nicht zwischen den verschiedenen AED-Typen bezogen auf defibrillierbare und nichtdefibrillierbare Herzrhythmen. Wegen der höheren Homogenität durch das Cross-over-Design (defibrillierbare und nichtdefibrillierbare Herzrhythmen in randomisierter Sequenz) war bei einer angenommenen Differenz *P*(X > Y) von 0,65 in der subjektiven Beurteilung der Verständlichkeit von AED-Sprachanweisungen eine Stichprobengröße von 59 ausreichend, um eine Effektstärke („power“) von 80 % und eine Signifikanz von 5 % zu erhalten (https://homepage.univie.ac.at/robin.ristl/samplesize.php?test=wilcoxon).

Die Datenanalyse wurde mit SPSS Statistics (Version 26, IBM, NY, USA) durchgeführt. Auf Normalverteilung wurde mit Shapiro-Wilk-Test überprüft. Nichtparametrische Tests wurden verwendet für die Evaluierung von ordinalen Daten und zum Vergleich zwischen zwei AED (Mann-Whitney-U-Test) sowie zwischen mehr als zwei AED (Kruskal-Wallis-Test). Die Signifikanzwerte des paarweisen Vergleichs der Defibrillatoren wurden mit der Bonferroni-Korrektur, zur Vermeidung der Alpha-Fehler-Kumulierung, angepasst.

## Ergebnisse

Insgesamt nahmen 72 Teilnehmer, davon 33 Männer (45,8 %) und 39 Frauen (54,2 %), an der Studie teil. Davon wurden 2 nicht in die Auswertung eingeschlossen (AED nicht eingeschaltet [*n* = 1], inkompletter Datensatz [*n* = 1]). Das mittlere Alter war 20,7 ± 3,4 Jahre.

12 (16,7 %) der Teilnehmer hatten keine Erfahrung mit Reanimationsmaßnahmen, 53 (73,6 %) hatten grundlegende Erfahrungen und 7 (9,7 %) einen fortgeschrittenen Erfahrungsgrad.

Bei 40 Szenarien mit initial defibrillierbarem Herzrhythmus wurde im Durchschnitt nach 125 s der erste Schock verabreicht. Bei 30 Notfallszenarien mit einem initial nichtdefibrillierbaren Herzrhythmus vergingen durchschnittlich 116 s bis zum Start der Herzdruckmassage. 61 (84,7 %) der Teilnehmer machten einen Notfallcheck, der Notruf wurde von 38 (52,7 %) abgegeben, Thoraxkompressionen während der Ladephase des AED von 13 (18,1 %) durchgeführt. Die Dauer der Sprachanweisungen bis zur zweiten Analyse variierte um 92,5 % zwischen 38,6 und 71,5 s in Abhängigkeit von Gerät und Hersteller (Tab. 1).

**Table 1:** 

AED	Textpassage		Dauer (s)
FR3	„Pads aufkleben. Pads-Stecker neben dem blinkenden Lämpchen einstecken.“		5,10
	„Patient nicht berühren! Analyse läuft. Patient nicht berühren!“		8,50
	*Nichtdefibrillierbarer Rhythmus*		
	„Kein Schock empfohlen! Herzdruckmassage und Beatmung beginnen.“		14,12^a^
	„Schock empfohlen! Patient nicht berühren! Schock jetzt abgeben! Blinkende, orangefarbene Taste jetzt drücken!“		21,03^a^
*–*		Gesamt:	48,75
HS1	„Alle Kleidungsstücke vom Brustkorb des Patienten entfernen. Falls nötig aufschneiden!“		6,18
	„Wenn der Brustkorb entblößt ist, die weiße Schutzfolie abziehen und die Elektroden herausnehmen.“		6,65
	„Elektrode wie abgebildet aufkleben. Fest auf den entblößten Brustkorb drücken.“		6,25
	„Patient nicht berühren! Herzrhythmus wird analysiert.“		4,85
	*Nichtdefibrillierbarer Rhythmus*		
	„Kein Schock empfohlen. Falls nötig Herdruckmassage und Beatmung beginnen.“		7,79
	*Defibrillierbarer Rhythmus*		
	„Schock empfohlen! Unbedingt Abstand zum Patienten halten! Blinkende, orangefarbene Taste jetzt drücken!“		20,43^a^
	„Schock verabreicht. Herzdruckmassage und Beatmung beginnen.“		6,64
–		Gesamt:	58,79
FE	„Setzen Sie die Reanimation fort und kleben Sie die Elektroden wie abgebildet auf den nackten und trockenen Oberkörper. Stecker beim gelben Licht einstecken.“		10,72^a^
	„Drücken Sie die blaue Taste.“		1,60
	„Patient nicht berühren! Bitte warten, Auswertung läuft.“		3,96
	*Nichtdefibrillierbarer Rhythmus*		
	„Kein Schock empfohlen! Führen Sie abwechselnd 30 Herzdruckmassagen und 2 Beatmungen durch. Setzen Sie die Reanimation fort, bis der Patient normal atmet oder neue Anweisungen erfolgen.“		28,29^a^
	*Defibrillierbarer Rhythmus*		
	„Schock empfohlen! Achtung, Patient nicht berühren! Gerät lädt.“	–	10,07^a^
	„Schock abgeben! Patient nicht berühren! Drücken Sie die orange Taste!“	–	4,61
	„Führen Sie abwechselnd 30 Herzdruckmassagen und 2 Beatmungen durch. Setzen Sie die Reanimation fort, bis der Patient normal atmet oder neue Anweisungen erfolgen.“	–	*12,20*^*a*^
–		Gesamt:	71,45
LP	„Elektroden anschließen.“		1,06
	„Elektrodenkontakt und Patientenanschluss überprüfen.“		2,96
	„Achtung, jetzt darf niemand den Patienten berühren! Zurücktreten! Herzrhythmus wird ausgewertet.“		7,16
	*Nichtdefibrillierbarer Rhythmus*		
	„Kein Schock empfohlen! Wiederbelebungsmaßnahmen durchführen! Beatmungen und Herzdruckmassagen durchführen!“		15,69^a^
	*Defibrillierbarer Rhythmus*		
	„Achtung, jetzt darf niemand den Patienten berühren! Blinkende Taste drücken!“		4,41
	„Wiederbelebungsmaßnahmen durchführen!“		
	„Beatmungen und Herzdruckmassagen durchführen!“		7,36
–		Gesamt:	38,64

^a^Dauer der Sprachanweisungen von mehr als 10 s

### HOI bei initial defibrillierbarem Rhythmus (Kammerflimmern)

Signifikante Unterschiede der HOI ergaben sich zwischen den vier eingesetzten AED für die Intervalle zwischen Herzrhythmusanalyse und Schockabgabe oder CPR (T6–T8; *p* = 0,002). Perischockpausen von unter 10 s konnten mit keinem der getesteten Geräte erreicht werden (Tab. 2).

**Table 2:** 

	FR3	HS1	FE	LP	*P*‑Wert
	m ± SD	m ± SD	m ± SD	m ± SD	
*Defibrillierbarer Herzrhythmus (Kammerflimmern)*					
T1–T5 (s)	86,8 ± 40,4	93,2 ± 27,8	91,0 ± 23,1	115,2 ± 69,0	0,800
T1–T6 (s)	88,9 ± 40,4	95,4 ± 28,2	96,9 ± 22,9	116,3 ± 69,4	0,752
T1–T8 (s)	105,2 ± 42,7	127,6 ± 44,7	120,9 ± 22,1	144,1 ± 67,3	0,428
T6–T7 (s)	11,0 ± 2,8	25,5 ± 23,6	9,7 ± 1,9	18,9 ± 6,0	<0,001
T6–T8 (s)	16,3 ± 4,1	32,2 ± 24,2	24,0 ± 4,1	27,8 ± 13,7	0,002
T7–T8 (s)	5,3 ± 2,8	6,7 ± 2,4	14,3 ± 3,8	8,9 ± 11,3	0,001
T9–T10 (s)	13,7 ± 7,6	NA	20,5 ± 4,2	9,9 ± 3,8	0,001
T9–T11 (s)	19,5 ± 7,8	NA	32,7 ± 5,4	22,0 ± 7,1	0,004
T10–T11 (s)	5,8 ± 1,8	NA	12,3 ± 4,4	12,1 ± 5,0	0,002
*Nichtdefibrillierbarer Herzrhythmus (Asystolie)*					
T1–T5 (s)	89,6 ± 17,5	NA	97,1 ± 20,0	83,5 ± 19,9	0,459
T1–T6 (s)	92,6 ± 18,1	NA	100,7 ± 20,7	84,9 ± 19,5	0,310
T1–T8 (s)	113,6 ± 18,3	NA	124,8 ± 17,0	108,1 ± 19,1	0,108
T6–T7 (s)	10,6 ± 2,3	NA	14,4 ± 4,7	13,9 ±7,8	0,342
T6–T8 (s)	21,1 ± 6,7	NA	24,2 ± 8,4	23,1 ± 12,6	0,804
T7–T8 (s)	10,5 ± 6,7	NA	9,7 ± 4,8	9,2 ± 5,5	0,823
T9–T10 (s)	11,4 ± 5,0	NA	13,2 ± 1,1	19,6 ± 9,6	<0,001
T9–T11 (s)	18,4 ± 5,0	NA	28,9 ± 3,6	24,7 ± 9,6	<0,001
T10–T11 (s)	7,0 ± 2,3	NA	15,7 ± 3,5	5,1 ± 1,6	<0,001

T1–T5: Start bis zum Aufkleben der Elektroden, T1–T6: Start bis zur ersten Sprachanweisung, T1–T8: Start bis zur ersten Schockabgabe/Herzdruckmassage, T6–T7: erste Herzrhythmusanalyse, T6–T8: erste Herzrhythmusanalyse bis zur ersten Schockabgabe/Herzdruckmassage, T7–T8: erste Schockabgabe/Herzdruckmassage bis zur Ausführung, T9–T10: zweite Herzrhythmusanalyse, T9–T11: zweite Herzrhythmusanalyse bis zur Schockabgabe/Herzdruckmassage, T10–T11: zweite Analyseempfehlung bis zur Ausführung

*NA* nicht auswertbar durch technische Panne

Ebenso unterschieden sich die HOI für Aufforderung bis Maßnahme (T7–T8; *p* = 0,001), Dauer der zweiten Herzrhythmusanalyse (T9–T10; *p* = 0,001), Beginn der zweiten Analyse bis zur Schockabgabe oder CPR (T9–T11; *p* = 0,004), zweite Aufforderung bis Maßnahme (T10–T11; *p* = 0,002; Tab. 2).

### HOI bei initial nichtdefibrillierbarem Rhythmus (Asystolie)

Signifikante Unterschiede gab es bei den Intervallen Dauer der zweiten Herzrhythmusanalyse (T9–T10; *p* < 0,001), Beginn der zweiten Analyse bis zur Schockabgabe oder CPR (T9–T11; *p* < 0,001) und der zweiten Aufforderung bis Maßnahme (T10–T11; *p* < 0,001; Tab. 2).

### Leichtigkeit der Anwendung und Zeitintervalle

Alle AED wurden vom Großteil der Anwender mit „sehr leicht“ und „leicht“ beurteilt (68,4–91,7 %). Weniger als ein Drittel bewertete den Umgang mit „mittel“ (8,3–31,6 %).

Nur ein AED wurde von 10,0 % der Anwender mit „schwierig“ beurteilt.

Zwischen FE, FR3 und LP konnten keine statistischen Unterschiede in der Notenverteilung bezüglich des Umgangs mit den Geräten festgestellt werden.

### Rückmeldungen und Verbesserungsvorschläge

Die Rückmeldungen der Probanden betrafen hauptsächlich die Markierung und Beschriftung der Geräte, Sprachanweisungen und Elektroden:

Schwierigkeiten mit dem Öffnen der Verpackung (HS1: *n* = 1).

Beschriftung am AED, mit welchem Schritt begonnen werden soll (FR3: *n* = 1; HS1: *n* = 1, FE: *n* = 2).

Unklarheit, ob das Gerät schon vor dem Kleben der Elektroden eingeschaltet werden muss (FR3: *n* = 6).

Erschwertes Auffinden der Klebeelektroden (FE: *n* = 4; LP: *n* = 4).

Die Abbildungen auf den Klebeelektroden zur richtigen Positionierung waren verwirrend (FR3: *n* = 2; HS1: *n* = 2; FE: *n* = 2; LP: *n* = 5).

Schwierigkeiten mit dem Abziehen der Folien von den Elektroden (FE: *n* = 1; LP: *n* = 1).

Konkretere Sprachanweisungen mit einer genaueren Anleitung wären sinnvoll (FR3: *n* = 3; HS1: *n* = 3; FE: *n* = 2; LP: *n* = 5).

#### Verbesserungsvorschläge.

Der Klettverschluss zum Öffnen des Defibrillators sollte mit einer auffälligen Farbe oder einem Pfeil deutlich markiert sein, damit klar ist, wo die Verpackung des Defibrillators zu öffnen ist und wie das Gerät einzuschalten ist.

Die Benutzeroberfläche sollte einheitlich gestaltet sein mit identischer Anordnung der Tasten und gleicher Beschriftung mit unterschiedlichen Farbtönen und mehr Leuchtkraft. Die Elektroden sollten nach dem Öffnen der Verpackung des AED auf den ersten Blick sichtbar sein. Die Abziehfolie der Elektroden sollte farblich markiert sein und der Schriftzug „Pull off“ sollte unmissverständlich auf das Abziehen der Folie hinweisen. Die Elektroden sollten bereits angeschlossen und einsatzbereit sein. Die Abbildungen auf den Klebeelektroden, welche die richtige Positionierung dieser auf dem Thorax darstellen, sollten größer aufgedruckt werden.

Die Knöpfe des Defibrillators mit Nummern in der Reihenfolge der Verwendung beschriften.

Mehr Sprachanweisungen für Ersthelfer zur Reanimation: eine Sprachanweisung zum Freimachen des Oberkörpers des Patienten, eine Sprachanweisung zum Verhältnis 30 Thoraxkompressionen zu 2 Beatmungen. Genauere Sprachanweisungen zur Handposition auf dem Brustkorb und zur Beatmung.

Ein akustischer Taktgeber für die Frequenz der Thoraxkompressionen.

Die Sprachanweisung „Patienten nicht berühren“ ergänzen mit „Analyse läuft“.

Die Sprachanweisung „Schock empfohlen“ ersetzen durch „Schock jetzt abgeben“.

## Diskussion

Die Verzögerung des Beginns der Herzdruckmassage, die Dauer der HOI während der CPR und die Anwenderzufriedenheit bei vier häufig verwendeten AED wurden untersucht. Die Zeit bis zur Einsatzbereitschaft des Geräts, ausgedrückt im Intervall T1–T5 von der Inbetriebnahme bis zum Aufkleben der Elektroden, war bei allen AED vergleichbar.

Im Gegensatz zu Untersuchungen von Muller et al. konnte mit allen vier AED die erste Defibrillation ohne signifikanten zeitlichen Unterschied erreicht werden [17].

HOI unterschieden sich in unserer Studie deutlich zwischen den verschiedenen AED-Typen, vor allem bei defibrillierbaren Herzrhythmen. Sprachanweisungen waren aber auch ursächlich für erhebliche HOI bei den nichtdefibrillierbaren Herzrhythmen.

Die „ERC guidelines“ empfehlen, die Perischockpausen unter 10 s zu halten [19]. Kürzere Perischockpausen stehen in signifikantem Zusammenhang mit höheren Überlebenschancen eines präklinischen Atemkreislaufstillstands mit defibrillierbaren Herzrhythmen [3]. Keines der bei dieser Arbeit getesteten AED-Geräte konnte das empfohlene Zeitlimit einhalten. Bereits bei den systemimmanenten Verzögerungen durch Sprachanleitungen gab es bei allen vier getesteten Geräten zumindest eine Textpassage, die länger als 10 s dauerte.

Zusätzliche HOI, die sich durch Beatmung über ein Beatmungstuch ergeben, fallen durch die aktuellen ERC-Empfehlungen aufgrund der COVID-19-Pandemie aus [18]. Um die HOI so kurz wie möglich zu halten, empfehlen die „ERC guidelines“ 2015 und 2020, die Pausen der Thoraxkompressionen für die Analyse des Herzrhythmus und während des Ladevorgangs des AED so gering wie möglich zu halten [4]. Man kann in der Ladephase ohne Verzögerung die Thoraxkompressionen weiterführen, aber dazu müssten die derzeitigen AED-Sprachanweisungen modifiziert werden [5]. Auch unterschiedliche Ladestrategien der AED, z. B. durch das Vorladen des Kondensators, bereits während der Herzrhythmusanalyse, könnten die HOI weiter verkürzen [21]. Einen neuen Ansatz bilden Algorithmen auf Basis von künstlichen neuronalen Netzwerken und Deep-Learning-Methoden, damit auch während der Herzrhythmusanalyse reanimiert werden kann, ohne die Qualität der Analyse zu beeinträchtigen [13, 14]. Bei Asystolie kann zum Beispiel durch einen vorgeschalteten Asystoliedetektor die Abwesenheit von elektrischen Herzaktivitäten identifiziert werden, ohne den kompletten Algorithmus durchlaufen zu müssen [13].

Vorschläge zur Erhöhung der Benutzerfreundlichkeit gehen von einer einheitlichen Benutzeroberfläche mit identischer Anordnung der Tasten und gleicher Beschriftung über automatisches Einschalten durch Öffnen der Verpackung des AED bis hin zur einfachen Auffindbarkeit und verbesserten Aufklebbarkeit der Elektroden. Wenn die Klebeelektroden bereits fix mit dem Kabel verbunden sind und erst durch Abziehen einer Klebefolie aktiviert werden, kann die entsprechende AED-Anweisung verkürzt werden („Elektroden aufkleben“ statt „Elektroden aufkleben und Stecker einstecken“). Die aufgedruckten Abbildungen auf den Klebeelektroden zur richtigen Positionierung auf dem Thorax sollten größer sein. Zahlreiche, aber auch zu kleine Abbildungen führen leicht zu Verunsicherungen über die richtige Elektrodenposition [6]. Inkorrektes Platzieren der Elektroden wird häufig berichtet [16]. Bereits geringe Abweichungen von der optimalen Position der Klebeelektroden können die Wirksamkeit der Defibrillation erheblich herabsetzen. Vor allem die apikale Elektrode wird von Laien oft zu weit kaudal platziert [8].

Automatisches Einschalten des AED, zum Beispiel durch die Entnahme von der Wandhalterung oder durch das Öffnen der Tasche, kann die Anwenderfreundlichkeit, und damit die Effizienz deutlich erhöhen. Das erspart auch Unsicherheiten, ob das Gerät zuerst eingeschaltet werden muss oder ob erst die Elektroden aufgeklebt werden müssen.

Einfaches Einschalten und Bedienen des AED erhöht auch die Wahrscheinlichkeit für die Durchführung von Reanimationsmaßnahmen durch Ersthelfer [16]. Mosesso et al. beobachteten, dass die häufigste Ursache für nicht durchgeführte Defibrillationen am Unvermögen lag, das Gerät einzuschalten [16].

Der Bedarf an ausführlicheren Sprachanweisungen hängt vom Erfahrungsgrad der Anwender ab. Erfahrene Anwender kennen die Abläufe und brauchen weniger Anweisungen als Personen mit wenig oder gar keiner Erfahrung [21]. Mindere Sprachqualität und geringe Lautstärke der Ansagen führen möglicherweise dazu, dass Anweisungen nicht durchgeführt werden und Reanimationsmaßnahmen verzögert werden [6]. Ein akustischer Taktgeber für Ersthelfer wäre sehr hilfreich, um die richtige Frequenz der Thoraxkompressionen zu finden. Insgesamt wurde jedoch allen getesteten Geräten eine hohe Benutzerfreundlichkeit attestiert.

### Limitationen

Die Studienpopulation war zwar homogen, da es sich bei den Teilnehmern um Medizinstudent*Innen des ersten Semesters handelte. Die Aussagekraft auf „public access defibrillation“ durch Laienhelfer ist damit eingeschränkt [22]. Es ist davon auszugehen, dass Medizinstudierende im Vergleich zur Allgemeinbevölkerung ein höheres Bildungsniveau und bei einer Laienreanimation eine höhere Motivation aufweisen und eher einschlägige Vorkenntnisse im Bereich der Wiederbelebung besitzen. Auch die experimentellen Versuchsbedingungen am Phantom sind nur bedingt vergleichbar mit der Reanimation eines Menschen bei einem echten Notfall. Auch waren die Reanimationsbedingungen einfacher, da die Phantome einfach zu entkleiden waren und z. B. auch keine Körperbehaarung aufwiesen, die gegebenenfalls noch zu entfernen gewesen wäre.

Für einen der vier von Rettungsorganisationen ausgeliehenen AED (Heartstart HS1; PHILIPS N.V. Healthcare, Amsterdam, Niederlande) konnten aus technischen Gründen (Akkumulatorladung) für initial nichtdefibrillierbare Herzrhythmen keine Untersuchungen gemacht werden. Auch für die zweite Herzrhythmusanalyse, Analyseempfehlung und Ausführung bei defibrillierbaren Herzrhythmen konnten keine Auswertungen gemacht werden.

## Fazit für die Praxis

Obwohl den getesteten AED eine hohe Benutzerfreundlichkeit attestiert wurde, dauerte es durchschnittlich 2 min bis zum Beginn der Herdruckmassage bzw. bis zur Abgabe des ersten Schocks. Keines der Modelle erreichte die geforderten HOI von unter 10 s. Zahlreiche Vorschläge, wie anwenderfreundliche Verpackung, übersichtliche Anordnung der Elektroden, leichtere Inbetriebnahme und erhöhte Präzision der Sprachanweisungen, könnten die Unterbrechungen der Herzdruckmassage weiter minimieren. Hilfreich sind auch unterschiedliche AED-Sprachanweisungsprotokolle für verschiedene Anwendergruppen, z. B. ausführlichere Anweisungen für Ersthelfer als für professionelle Helfer. Um die Handhabung der AED weiter zu verbessern, wird es aber notwendig sein, HOI rund um Analyse, Ladung und Schockabgaben auf ein Minimum zu verkürzen, indem die Sprachanweisungen unterschiedlicher AED möglichst einheitlich, unmissverständlich, kurz und präzise gehalten werden.

## References

[1] Beesems SG, Berdowski J, Hulleman M, Blom MT, Tijssen JG, Koster RW (2016). Minimizing pre- and post-shock pauses during the use of an automatic external defibrillator by two different voice prompt protocols. A randomized controlled trial of a bundle of measures. Resuscitation.

[2] Blom MT, Beesems SG, Homma PCM, Zijlstra JA, Hulleman M, van Hoeijen DA (2014). Improved survival after out-of-hospital cardiac arrest and use of automated external defibrillators. Circulation.

[3] Cheskes S, Schmicker RH, Verbeek PR, Salcido DD, Brown SP, Brooks S (2014). The impact of peri-shock pause on survival from out-of-hospital shockable cardiac arrest during the Resuscitation Outcomes Consortium PRIMED trial. Resuscitation.

[4] Cheskes S, Common MR, Byers PA, Zhan C, Morrison LJ (2014). Compressions during defibrillator charging shortens shock pause duration and improves chest compression fraction during shockable out of hospital cardiac arrest. Resuscitation.

[5] de Gauna SR, Irusta U, Ruiz J, Ayala U, Aramendi E, Eftestol T (2014). Rhythm analysis during cardiopulmonary resuscitation: past, present, and future. Biomed Res Int.

[6] Fleischhackl R, Roessler B, Domanovits H, Singer F, Fleischhackl S, Foitik G (2008). Results from Austria’s nationwide public access defibrillation (ANPAD) programme collected over 2 years. Resuscitation.

[7] Fleischhackl R, Losert H, Haugk M, Eisenburger P, Sterz F, Laggner AN (2004). Differing operational outcomes with six commercially available automated external defibrillators. Resuscitation.

[8] Foster AG, Deakin CD (2019). Accuracy of instructional diagrams for automated external defibrillator pad positioning. Resuscitation.

[9] Garza AG, Gratton MC, Salomone JA, Lindholm D, McElroy J, Archer R (2009). Improved patient survival using a modified resuscitation protocol for out-of-hospital cardiac arrest. Circulation.

[10] Gmb HNN (2020) Definetzwerk. https://definetzwerk.at. Zugegriffen: 2. Mai 2020

[11] Hansen CM, Rosenkranz SM, Folke F, Zinckernagel L, Tjornhoj-Thomsen T, Scient M (2017). Lay bystanders’ perspectives on what facilitates cardiopulmonary resuscitation and use of automated external defibrillators in real cardiac arrests. JAHA.

[12] Hedges JR, Sehra R, Van Zile JW, Anton AR, Bosken LA, O’Connor RE (2006). Automated external defibrillator program does not impair cardiopulmonary resuscitation initiation in the public access defibrillation trial. Acad Emerg Med.

[13] Irusta U, Ruiz J, Aramendi E, de Gauna SR, Ayala U, Alonso E (2012). A high-temporal resolution algorithm to discriminate shockable from nonshockable rhythms in adults and children. Resuscitation.

[14] Isasi I, Irusta U, Aramendi E, Eftestol T, Kramer-Johansen J, Wik L (2020). Rhythm analysis during cardiopulmonary resuscitation using convolutional neural networks. Entropy.

[15] Likert R (1932). A technique for the measurement of attitudes. Arch Psychol.

[16] Mosesso VN, Shapiro AH, Stein K, Burkett K, Wang H (2009). Effects of AED device features on performance by untrained laypersons. Resuscitation.

[17] Muller MP, Poenicke C, Kurth M, Richter T, Koch T, Eisold C (2015). Quality of basic life support when using different commercially available public access defibrillators. Scand J Trauma Resusc Emerg Med.

[18] Nolan JP, Monsieurs KG, Bossaert L, Bottiger BW, Greif R, Lott C (2020). European Resuscitation Council COVID-19 guidelines executive summary. Resuscitation.

[19] Perkins GD, Handley AJ, Koster RW, Castren M, Smyth MA, Olasveengen T (2015). European resuscitation council guidelines for resuscitation 2015 section 2. adult basic life support and automated external defibrillation. Resuscitation.

[20] Plattner R, Schabauer W, Baubin MA, Lederer W (2013). Hands-off time by AED voice prompts. Notfall Rettungsmed.

[21] Rhee JE, Kim T, Kim K, Choi S (2009). Is there any room for shortening hands-off time further when using an AED?. Resuscitation.

[22] WMA declaration of Helsinki—ethical principles for medical research involving human subjects. https://www.wma.net/policies-post/wma-declaration-of-helsinki-ethical-principles-for-medical-research-involving-human-subjects/ (Erstellt: 9. Juli 2018). Zugegriffen: 03.04.2022

